# Spin Noise Detection of Nuclear Hyperpolarization at 1.2 K

**DOI:** 10.1002/cphc.201500805

**Published:** 2015-11-13

**Authors:** Maria Theresia Pöschko, Basile Vuichoud, Jonas Milani, Aurélien Bornet, Matthias Bechmann, Geoffrey Bodenhausen, Sami Jannin, Norbert Müller

**Affiliations:** [a] Institute of Organic Chemistry, Johannes Kepler University LinzAltenbergerstraße 69, 4040, Linz, Austria E-mail: norbert.mueller@jku.at; [b] Institut de Sciences et Ingénerie Chimiques, Ecole Polytechnique Fédérale de Lausanne1015, Lausanne, Switzerland E-mail: sami.jannin@epfl.ch; [c] Département de Chimie, Ecole Normale Supérieure, PSL24 Rue Lhomond, 75231, Paris, Cedex 05, France; [d] Université Pierre-et-Marie Curie4 Place Jussieu, 75005, Paris (France; [e] UMR 7203, CNRS/UPMC/ENS, Ecole Normale SupérieureParis, France; [f] Bruker BioSpin AGIndustriestrasse 26, 8117, Fällanden, Switzerland; [g] Faculty of Science, University of South BohemiaBranišovská 1645/31A, 370 05, České Budějovice, Czech Republic

**Keywords:** dynamic nuclear polarization, non-linear effects, nuclear magnetic resonance, radiation damping, spin noise

## Abstract

We report proton spin noise spectra of a hyperpolarized solid sample of commonly used “DNP (dynamic nuclear polarization) juice” containing TEMPOL (4-hydroxy-2,2,6,6-tetramethylpiperidine *N*-oxide) and irradiated by a microwave field at a temperature of 1.2 K in a magnetic field of 6.7 T. The line shapes of the spin noise power spectra are sensitive to the variation of the microwave irradiation frequency and change from dip to bump, when the electron Larmor frequency is crossed, which is shown to be in good accordance with theory by simulations. Small but significant deviations from these predictions are observed, which can be related to spin noise and radiation damping phenomena that have been reported in thermally polarized systems. The non-linear dependence of the spin noise integral on nuclear polarization provides a means to monitor hyperpolarization semi-quantitatively without any perturbation of the spin system by radio frequency irradiation.

## 1. Introduction

Hyperpolarization by dissolution dynamic nuclear polarization (D-DNP)[1] has become a method of choice for the preparation of spin systems in highly polarized states, exceeding thermal polarization levels by four to five orders of magnitude. The greatly enhanced NMR sensitivity brought about by D-DNP has opened the way to exciting new applications, such as real-time metabolic imaging and localized spectroscopy for the detection of tumors in humans.[2] One important practical aspect of D-DNP is the observation of NMR signals at low temperatures, usually around 1.2 K. This step is essential for the optimization of DNP, which depends on numerous parameters such as sample formulation, choice of polarizing agents, microwave frequency irradiation, and so forth. The NMR signal observation is commonly performed by pulsed Fourier transform NMR, which unfortunately causes a loss of the polarization even when using pulses with small nutation angles. In contrast to pulsed NMR, spin noise NMR detection provides information on spin systems without any perturbation.[3–7]

The detection of spin noise is intimately linked with radiation damping (RD), in particular at high polarization levels, where RD becomes a non-negligible effect. RD leads to intricate dependencies of the line shapes on various parameters of the sample and the radio-frequency (rf) circuit used for detection.[8,9] Spin noise spectra of hyperpolarized liquid samples have been reported before on ^129^Xe[10] and in DNP experiments on ^1^H *after* dissolution at room temperature.[11,12]

In this communication we explore the potential of ^1^H spin noise spectroscopy for monitoring nuclear polarization states during DNP experiments before dissolution at 1.2 K. We show that spin noise detection provides a way to monitor the effect of the microwave frequency on DNP (both positive and negative polarizations) without perturbation by rf-irradiation. The optimum tuning of the probe (here 250 kHz below the nuclear Larmor frequency) can also be determined without perturbation.

## 2. Results

### 2.1. Effects of Polarization on Spin Noise

**Figure 1:**
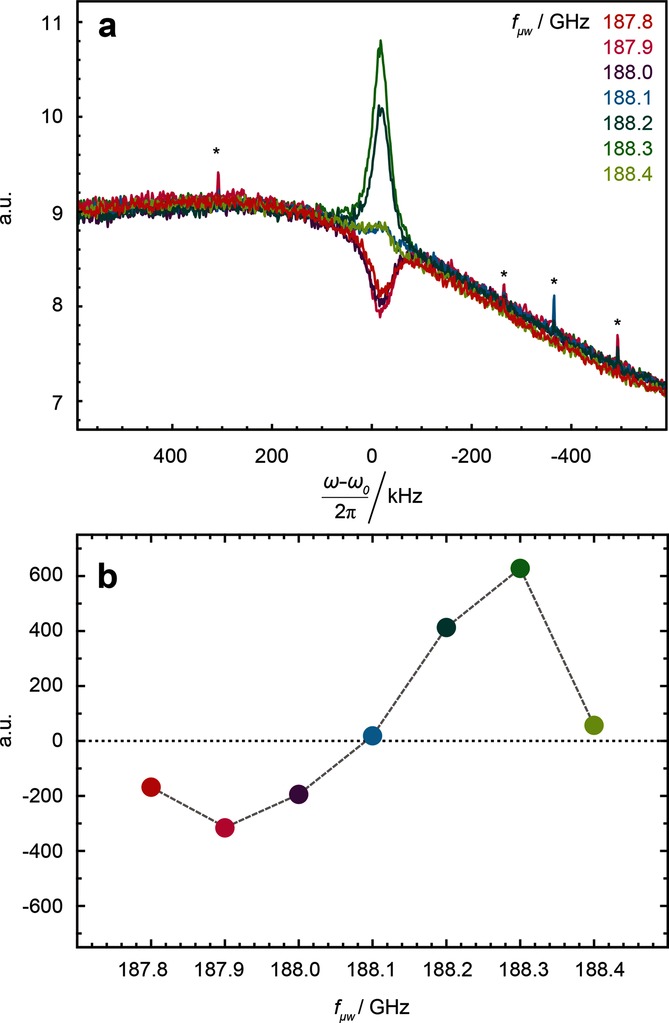
a) Proton (^1^H) spin noise power spectra, and b) corresponding integrals of these spectra as a function of the microwave irradiation frequency *f*_μw_ . Asterisks in (a) indicate artifacts stemming from intermittent pick-up from external, unidentified rf sources. In the absence of spin noise the thermal noise gives rise to a non-vanishing baseline.

When applying microwave irradiation, the ^1^H polarization *P*(^1^H) is enhanced by DNP, so that spin noise signals emerge from the background of thermal noise. As usual, the amplitude and sign of the polarization *P*(^1^H) depends on the position of the microwave irradiation frequency relative to the EPR transitions.[1] As shown in Figure 1, a positive polarization *P*(^1^H)>0, which can be induced by microwave irradiation below the electron Larmor frequency, leads to a decrease in noise power (i.e. a dip in the power spectrum below the level of the thermal circuit noise) while a negative polarization *P*(^1^H)<0, which can be induced by applying the microwave irradiation above the electron Larmor frequency, leads to an increase (i.e. a bump) in the power spectrum above the level of the thermal circuit noise. This is in accordance with the theory by McCoy and Ernst[8] as discussed in some detail below.

### 2.2. The Influence of Radio Frequency Tuning

**Figure 2:**
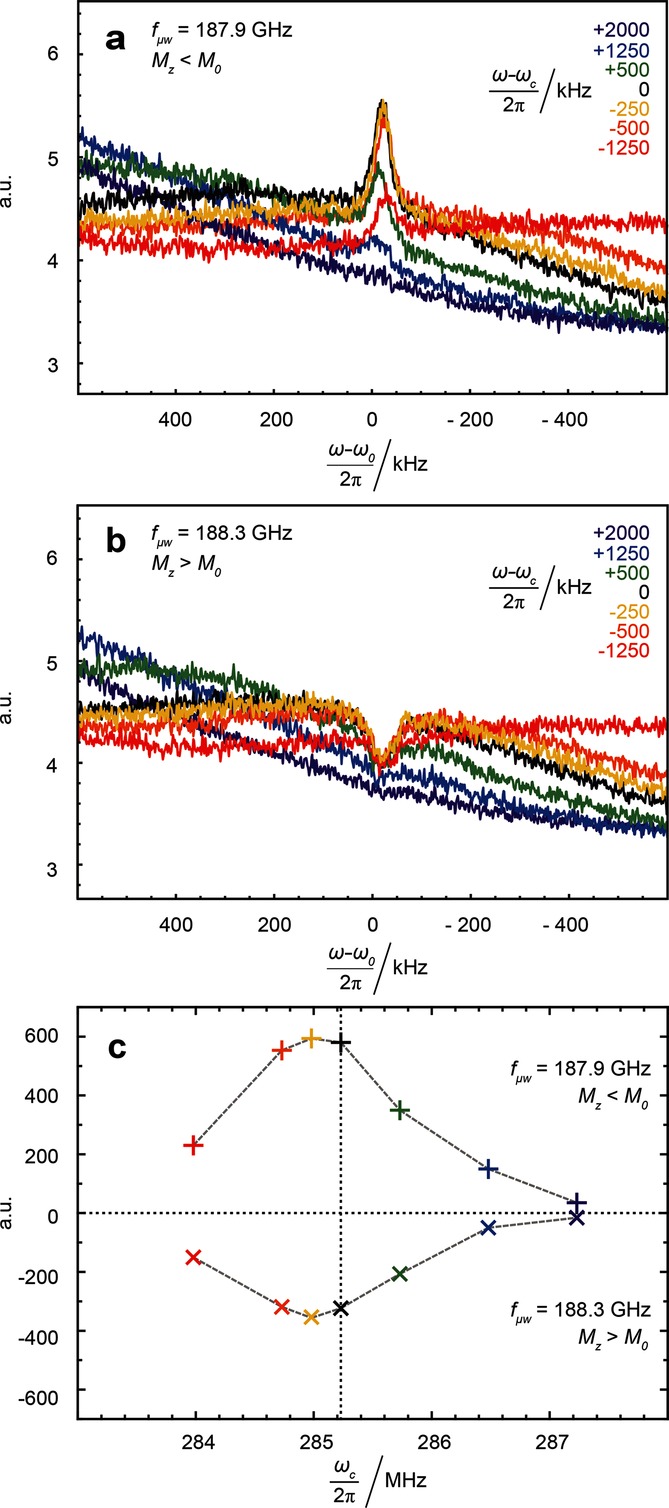
Noise power spectra (a,b) and noise power signal integrals (c) for different rf-tuning positions of the minimum of the wobble curve with respect to the SNTO. Microwave irradiation frequencies: 187.9 GHz (a and upper trace in c) and 188.3 GHz (b and lower trace in c). The colors indicate the corresponding offsets of the tuning frequencies with respect to the Larmor frequency *ω*_0_ of the protons at 6.7 T.

We systematically varied the resonance frequency of the rf-detector coil in the ^1^H range by adjusting the tuning capacitor. The dependence of the spin noise spectrum on rf-tuning is shown in Figure 2 when irradiating at either of two microwave frequencies 188.3 or 187.9 GHz, which yield the largest positive bump and negative dip in the spin noise power signals in Figure 1 b. In accordance with previous observations[9,13,14] the highest spin noise amplitudes are observed when the NMR probe circuit is significantly mistuned, a phenomenon not covered by the theory of McCoy and Ernst.[8] Both for positive and negative proton polarization, this mistuning offset amounts to −250 kHz.

## 3. Discussion

### 3.1. Spin Noise Line Shapes

The dependence of experimental spin noise line shapes on the polarization and tuning can be described by the theory of McCoy and Ernst.[8] Starting from a RLC circuit model with Nyquist noise voltages for both the circuit and the spins and assuming equal temperatures for both, they derived the following formula for the noise voltage spectral density [Eq. (1)]:


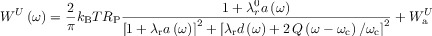
(1)

with the Boltzmann constant *k*_B_, the temperature *T*, the equivalent parallel resistance *R*_p_ of the circuit, the radiation damping rate at thermal equilibrium 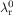, the actual radiation damping rate *λ*_r_ , the probe quality factor *Q*, the receiver circuit resonance frequency *ω*_c_, the noise voltage from sources external to the resonant circuit 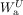
as well as the absorptive *a* and dispersive *d* components of the Lorentzian signal centered at the proton Larmor frequency *ω*_0_ [Eq. (2)]:


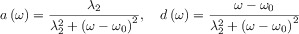
(2)

Thus, the appearance of line shapes due to spin noise predominantly depends on the line width *λ*_2_ (half width at half height), the offset (*ω*−*ω*_c_)/*ω*_c_ from the Spin Noise Tuning Optimum (SNTO), and the radiation damping (RD) rate 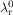
for thermal equilibrium polarization [Eq. (3)]:


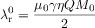
(3)

The equilibrium magnetization *M*_0_ of a system comprising *n* spins in thermal equilibrium is given by Equation (4):


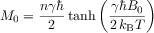
(4)

When the magnetization *M*_z_ deviates from its thermal equilibrium *M*_0_ (e.g. is attenuated through presaturation as discussed in Ref. [8], or enhanced through hyperpolarization, as discussed here), its amplitude can be described by an attenuation or enhancement factor *K*=*M*_z_/*M*_0_.[10] The longitudinal magnetization *M*_z_ affects the power line shape that arises from spin noise through the radiation damping rate *λ*_r_ [Eq. (5)]:


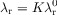
(5)

Note that while the fluctuations of the transverse components of magnetization depend only on the total number of spins, 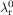
depends on the equilibrium magnetization as *λ*_r_ is proportional to the total polarization *P*(^1^H).[3,8,11]

If (*ω*−*ω*_c_=0) and (*ω*−*ω*_0_=0), that is, if the offset of the tuning maximum with respect to the SNTO and the offset with respect to the Larmor resonance frequency *ω*_0_ both vanish, Equation (1) can be simplified to Equation (6):


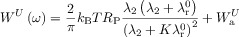
(6)

Equations (1) and (6) do not provide an explanation for the experimental observation that the largest signals were obtained at 285.23 MHz, when the circuit is tuned to about 250 kHz below the Larmor frequency (Figure 2). This offset corresponds to the SNTO position.[9,13–15]

It should be noted that no tuning dependence of the spin noise line shape was observed within the accessible tuning range. This is in contrast to previous experiments on thermal polarization using cryogenically cooled high-resolution liquid-state NMR probes.[9,13]

In Figure 1 the thermal noise power level is not constant over the entire spectral width, a feature that could easily be mistaken for an instrumental artifact. The curvature of the thermal power level curve is caused by Nyquist noise,[16,17] which is further corroborated by the tuning-dependent baseline changes seen in Figures 2 a,b.

An expression for the noise power spectral density of the background circuit can easily be derived from Equation (1) by setting the magnetization and hence the radiation damping rates 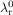
and *λ*_r_ to zero. The thermal noise base line is then described by Equation (7):


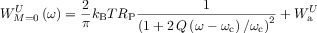
(7)

In principle, this equation defining the baseline shape can be used to determine the quality factor *Q* of the rf-reception circuit.

### 3.2. Quantitative Aspects

It appears useful to determine the enhancement factor *K* from spin noise spectra. Provided the conditions of Equation (6) are fulfilled, the full line width at half height in units of Hz is given by Equation (8):


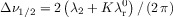
(8)

Thus, the enhancement factor *K* can in principle be estimated from the line width.[10] In our case however, the resolution of the experimental spin noise power spectra is insufficient to determine the line width with sufficient accuracy.

Alternatively, as has been demonstrated before,[10] *K* can also be derived from the integral of the spin noise power signal after subtracting the thermal noise baseline 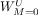
(*ω*) given in Equation (7), to obtain Equation (9):


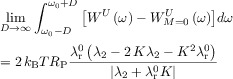
(9)

In both cases, prior knowledge of *λ*_2_ and of 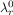
is required. If *λ*_2_≫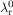, *λ*_2_ can be estimated from the line width of pulse spectra recorded during early stages of the hyperpolarization build-up. However, 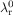
cannot be extracted from the same spectrum. Indeed, if the thermal line width is dominated by *λ*_2_, a small error on *λ*_2_ causes a large error on 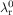. Thus one has to rely on the calculation of 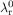
from Equation (3), which requires exact knowledge of the filling factor *η* and the quality factor *Q*. Errors in these parameters will of course affect the estimate of *K*. In the Supporting Information we discuss the non-linear dependence of the spin noise power integral on the enhancement factor *K*. As the shape of the function *K* versus *M*_z_ critically depends on 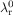, quantitative evaluation will require an independent accurate determination of *η* and *Q*.

**Figure 3:**
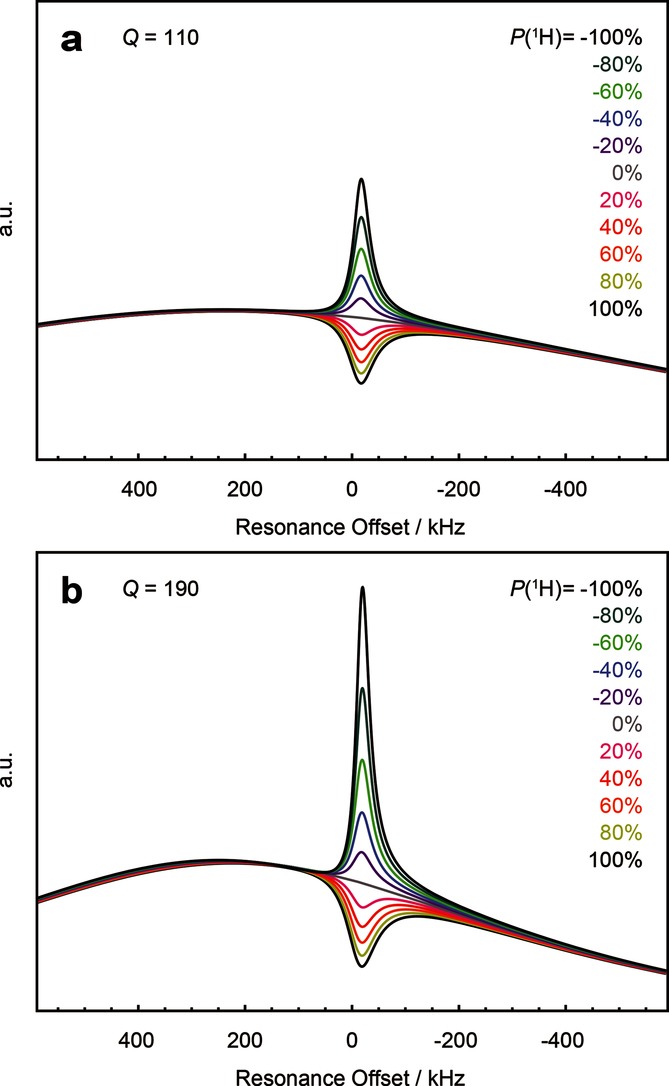
Computed spin noise power spectra according to Equation (1) for different polarization levels (+100 %>*P*(^1^H)>−100 %) as indicated by the color code in the legend. For thermal polarization at 1.2 K and 6.7 T *P*(^1^H)=0.6 %, which, at this scale and under our experimental uncertainties, is indistinguishable from the curve for *P*(^1^H)=0.0 %. An offset (*ω*−*ω*_c_)/2 π=250 kHz of the tuning maximum with respect to the spin noise tuning optimum (SNTO), an offset (*ω*−*ω*_0_)/2 π=−17 kHz with respect to the Larmor resonance frequency, and an intrinsic line width *λ*_2_/π=51 kHz are assumed. The probe quality factors used are *Q*=110 (a) and 190 (b). Radiation damping rates at thermal equilibrium 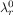
were calculated from Equations (3) and (4) using *η*=0.05, *T*=1.2 K, and *B*_0_=6.7 T with *M*_0_ corresponding to a spin number density *n*=3.11×10^27^ spins m^−3^. Note that a quality factor *Q*=190 was experimentally determined from the tuning curve, although the baselines computed for *Q*=110 [Eq. (7)] match the baselines in the experimental spin noise spectra (Figure 1 a) more closely, as discussed in the text.

For this reason we cannot achieve unambiguous fits to the experimental data here. The theoretical spin noise power spectra shown in Figure 3 were computed using Equation (1) with parameters corresponding closely to our experimental setup.

Visual inspection shows a remarkable agreement between the calculated (Figure 3) and experimental spectra (Figure 1). Quantitative fits to the experimental data are in principle possible, but would require a priori knowledge of important parameters such as the polarization *P*(^1^H), the filling factor *η*, and the quality factor *Q* of the probe circuit.

The grey trace in Figure 3 corresponds to the baseline given by Equation (7). It is evident from Figures 2 a,b that our home-built probe[18] shows some deviations from the predicted behavior. Comparing the baselines of the simulated spectra in Figure 3 to the experimental ones, it appears that a value *Q*=110 reproduces the baseline curvature better than the experimentally determined value *Q*=190. Such a divergence of quality factor values is not unusual since the rf-circuit relevant for detection of spin noise includes the cables and the pre-amplifier and the crossed diodes are in a different state than during rf-excitation, which is used in the tuning (“wobbling”) procedure.[9,19,20] In most previous spin noise experiments the change in baseline was not visible, since only narrower spectral widths were studied.

## 4. Conclusions

We have shown that the observation of ^1^H spin noise spectra of typical “DNP juice” during microwave irradiation at 1.2 K allows one to monitor the nuclear polarization levels *P*(^1^H) without interference due to rf-irradiation. While at microwave irradiation frequencies above the electron Larmor frequency the usual dip-shaped (i.e. negative) spin noise signal is observed, a positive bump occurs when the polarization *P*(^1^H) is negative, as can be achieved with microwave irradiation at frequencies below the electron Larmor frequency. These experimental results corroborate the behavior predicted by the theory of spin noise line shapes by McCoy and Ernst.[8] To our knowledge this is the first study of nuclear magnetic spin noise at liquid helium temperature and also represents the first investigation of spin noise from a hyperpolarized solid with negative polarization. It complements and extends previous observations of spin noise signals using cryogenically cooled probes and thermal samples at ambient temperature[7,9,13,14,19] as well as hyperpolarized samples at ambient temperature.[10–12] Our experiments and simulations also suggest that spin noise detection could be used for the quantitative determination of polarization levels without rf-irradiation, provided the filling factor and the quality factor are known. With the current setup, it is possible to monitor the build-up of the polarization *P*(^1^H) in a semi-quantitative manner. With an optimized detection pathway providing higher “spin-noise-to-thermal-noise” ratios, real-time monitoring of slow DNP build-up during microwave irradiation should be feasible. Further experimental work is in progress.

## Experimental Section

Spin noise power spectra were acquired using a home-built polarizer and probe described previously[18,21–23] using a 6.7 T magnet equipped with a continuous flow helium cryostat and a doubly tuned (^1^H and ^13^C) saddle–coil probe with a proton resonance frequency of 285.23 MHz. Continuous-wave microwave irradiation was performed at frequencies 187.5>*f*_μw_>188.5 GHz with a power *p*_μw_≈80 mW. The quality factor of the NMR circuit was estimated to be *Q*≈190 from the width of the rf-tuning curve. 25 frozen beads (∼10 μL each) of a commonly used solution (“DNP juice”) containing 20 % H_2_O, 30 % D_2_O, 50 % glycerol-*d*_8_, and 50 mm TEMPOL were placed in an rf*-*coil with an active volume of ∼1.0 mL. At 1.2 K the full line-width at half height of the ^1^H signal was estimated from a conventional spectrum to be about 51 kHz, measured using a small flip angle pulse during the build-up of the hyperpolarization after presaturation.

All spin noise spectra were measured at DNP equilibrium, that is, after the polarization had reached steady state. Under microwave irradiation these steady states are typically reached with a time constant *τ*_DNP_(^1^H)=20–180 s, which can slightly depend on the microwave irradiation frequency. In our DNP conditions these so-called DNP build-up times *τ*_DNP_ are usually much shorter than the proton spin lattice relaxation times, typically of the order of *T*_1_(^1^H)=2000 s. In addition, the build-up time constants for the positive and negative polarizations were determined to be 71 s and 114 s, respectively, from the line widths in small flip angle pulse spectra during microwave irradiation following the approach by Desvaux et al.[10] Spin noise data in this communication were always recorded more than 600 s after the last rf-pulse applied to the sample.

To record spin noise spectra, blocks of 1024 complex data points were acquired with a spectral width of 1.25 MHz centered on the proton Larmor frequency. For each microwave irradiation frequency, 65 536 noise blocks were recorded giving a total acquisition time of about 26 min for each spectrum. For each rf-tuning frequency of the proton channel, 8192 noise blocks were acquired. The power spectra were added according to the procedure described by Nausner et al.[13]

A 5^th^-order polynomial base line correction was applied before evaluating noise power signal integrals. Subtracting the baseline in this way allows one to remove broadband noise stemming from Nyquist noise of the coil and distortions caused by the filters of the spectrometer. This ensures that the line shapes and their integrals reflect only contributions to the noise spectrum that originate from the nuclear spins.

It should be noted that we did not optimize our instrumental setup for the acquisition of spin noise spectra. Without DNP, that is, with thermal polarization at 1.2 K, we could not observe any spin noise with the sample and conditions described above. Using state-of-the-art low-noise pre-amplifiers and low-noise electronics, it should be possible to significantly reduce the accumulation times required to observe spin noise in the future.

## Supporting Information

As a service to our authors and readers, this journal provides supporting information supplied by the authors. Such materials are peer reviewed and may be re-organized for online delivery, but are not copy-edited or typeset. Technical support issues arising from supporting information (other than missing files) should be addressed to the authors.

## References

[b1] Abragam A, Goldman M (1978). Rep. Prog. Phys.

[2] Nelson SJ, Kurhanevicz J, Vigneron DB, Larson PEZ, Harzstark AL, Ferrone M, van Criekinge M, Chang JW, Bok R, Park I, Reed G, Carvajal L, Small EJ, Munster P, Weinberg VK, Arkandjaer-Larsen JH, Chen AP, Hurd RE, Odegardstuen L-I, Robb FJ, Tropp J, Murray JA (2013). Sci. Transl. Med.

[3] Bloch F (1946). Phys. Rev.

[4] Müller N, Jerschow A (2006). Proc. Natl. Acad. Sci. USA.

[5] Degen CL, Poggio M, Mamin HJ, Rugar D (2007). Phys. Rev. Lett.

[6] Jurkiewicz A (2015). Chem. Phys. Lett.

[7] Müller N, Jerschow A, Schlagnitweit J, Griffiths JR, Young IR, Wasylishen RE (2013). eMagRes., Vol. 2.

[8] McCoy M, Ernst R (1989). Chem. Phys. Lett.

[9] Pöschko MT, Schlagnitweit J, Huber G, Nausner M, Horničaková M, Desvaux H, Müller N (2014). ChemPhysChem.

[10] Desvaux H, Marion DJY, Huber G, Berthault P (2009). Angew. Chem. Int. Ed.

[01] (2009). Angew. Chem.

[11] Giraudeau P, Müller N, Jerschow A, Frydman L (2010). Chem. Phys. Lett.

[12] Chen H-Y, Lee Y, Bowen S, Hilty C (2011). J. Magn. Reson.

[13] Nausner M, Schlagnitweit J, Smrečki V, Yang X, Jerschow A, Müller N (2009). J. Magn. Reson.

[14] Nausner M, Goger M, Bendet-Taicher E, Schlagnitweit J, Jerschow A, Müller N (2010). J. Biomol. NMR.

[15] Marion DJ-Y, Desvaux H (2008). J. Magn. Reson.

[16] Nyquist H (1928). Phys. Rev.

[17] Sleator T, Hahn E, Hilbert C, Clarke J (1985). Phys. Rev. Lett.

[18] Comment A, van den Brandt B, Uffmann K, Kurdzesau F, Jannin S, Konter J, Hautle P, Wenckebach W, Gruetter R, van der Klink J (2007). Concepts Magn. Reson. Part B.

[19] Bendet-Taicher E, Müller N, Jerschow A (2014). Concepts Magn. Reson. Part B.

[20] Ferrand G, Huber G, Luong M, Desvaux H (2015). J. Chem. Phys.

[21] Bornet A, Milani J, Wang S, Mammoli D, Buratto R, Salvi N, Segawa TF, Vitzthum V, Miéville P, Chinthalapalli S, Perez-Linde AJ, Carnavale D, Jannin S, Caporini M, Ulzega S, Rey M, Bodenhausen G (2012). Chimia.

[22] Bornet A, Melzi R, Linde AJP, Hautle P, van den Brandt B, Jannin S, Bodenhausen G (2013). J. Phys. Chem. Lett.

[23] Vuichoud B, Milani J, Bornet A, Melzi R, Jannin S, Bodenhausen G (2014). J. Phys. Chem. B.

