# Supplementary material for: Spin Noise Detection of Nuclear Hyperpolarization at 1.2 K
Source: Chemphyschem. 2015 Nov 13;16(18):3859–64. doi: 10.1002/cphc.201500805 (PMC4691331; doi:10.1002/cphc.201500805)
Supplement: Supplementary file 1 — Supplementary [file cphc0016-3859-sd1.pdf]

CHEMPHYSCHEM

Supporting Information

Spin Noise Detection of Nuclear Hyperpolarization at 1.2 K

Maria Theresia Pöschko,[a] Basile Vuichoud,[b] Jonas Milani,[b] Aurélien Bornet,[b]
Matthias Bechmann,[a] Geoffrey Bodenhausen,[b, c, d, e] Sami Jannin,*[b, f] and
Norbert Müller*[a, g]

cphc_201500805_sm_miscellaneous_information.pdf

Supporting Information

Quantitative aspects

The influence of Q and η on λ_r^0

To calculate the enhancement factor K according to Equations (8) or (9) of the main text, the thermal equilibrium radiation damping rate λ_r^0 is required. In Table S1, λ_r^0 is calculated for a range of η and Q values for common experimental conditions using Eq. (4) in the main text. There is a deviation by a factor of 80 between the smallest and the largest calculated values.

Table S1: Calculated radiation damping rates λ_r^0 in thermal equilibrium for temperature $T = 1.2\ \mathrm{K}$, magnetic field strength $B_0 = 6.7$ T and a spin number density n = $3.33\text{x}10^{27}$ m^{-3} for different values of the quality factor Q and the filling factor η. Colored numbers correspond to the ones used in Table S2 and the traces in Figs. S1 and S2.

		Q						
	λ_r^0 / rad s^{-1}	70	90	110	130	150	170	190
η	0.05	**157.7**	202.8	**247.8**	292.9	337.9	383.0	**428.0**
	0.10	315.4	405.5	495.6	585.7	675.9	766.0	**856.1**
	0.15	473.1	608.3	743.4	878.6	1013.8	1149.0	**1284.1**

Computation of the enhancement factor K from the line width $\Delta\nu_{1/2}$ and the radiation damping rate λ_r^0

For this purpose, the line widths of spectra excited by small flip angle pulses under steady state DNP conditions were determined at the microwave irradiation frequencies yielding the largest positive polarization (187.9 GHz, $\Delta\nu_{1/2} = 60\ \mathrm{kHz}$, i.e. spin noise dip), and at the largest negative polarization (188.3 GHz, $\Delta\nu_{1/2} = 41\ \mathrm{kHz}$, i.e. spin noise bump). In Fig. S1, the calculated full line widths at half maximum $\Delta\nu_{1/2}$ are drawn as a function of K for different λ_r^0.

In Table S2, enhancement factors K calculated from the observed line widths and three different radiation damping rates λ_r^0 are displayed. As expected from visual inspection of Figure S1, the computed enhancement factors strongly depend on λ_r^0.

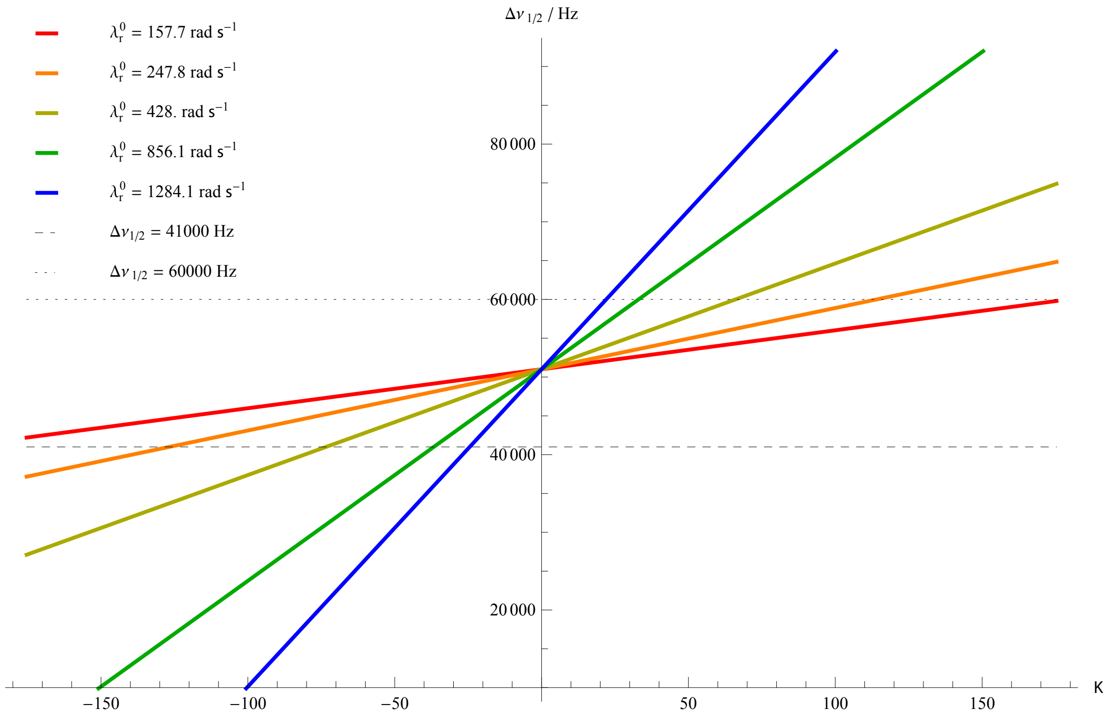

Figure S1: Full line width at half height $\Delta\nu_{1/2}$ calculated from Eq. (8) in the main text as a function of the enhancement factor K for five different values of the radiation damping rate λ_r^0 with the assumptions of perfect tuning $(\omega - \omega_c) = 0$, vanishing resonance offset $(\omega - \omega_0) = 0$ and a line width $\lambda_2/\pi = 51$ kHz. The smallest and largest line widths observed in pulse spectra are indicated by the dashed and dotted lines, respectively. The colors match with the corresponding entries in Tables S1 and S2.

Table S2: Enhancement factors K calculated according to Eq. (8) from the smallest and largest experimental line width (from spectra excited by short pulses) and three different radiation damping rates λ_r^0.

$f_{\mu w}$ / GHz	$\Delta\nu_{1/2}$ / kHz	K		
		λ_2/π= 51 kHz<br>λ_r^0 = **157.7** rad s^{-1}	λ_2/π= 51 kHz<br>λ_r^0 = **856.1** rad s^{-1}	λ_2/π= 51 kHz<br>λ_r^0 = **1284.1** rad s^{-1}
187.9 (pos. Pol.)	60	179.2	33.0	22.0
188.3 (neg. Pol.)	41	-199.2	-36.7	-24.5

Dependence of the noise power integral on the enhancement factor K

In Figure S2, the dependence of the spin noise integral on the enhancement factor K computed from Eq. (9) in the main text is shown for different values of λ_r^0.

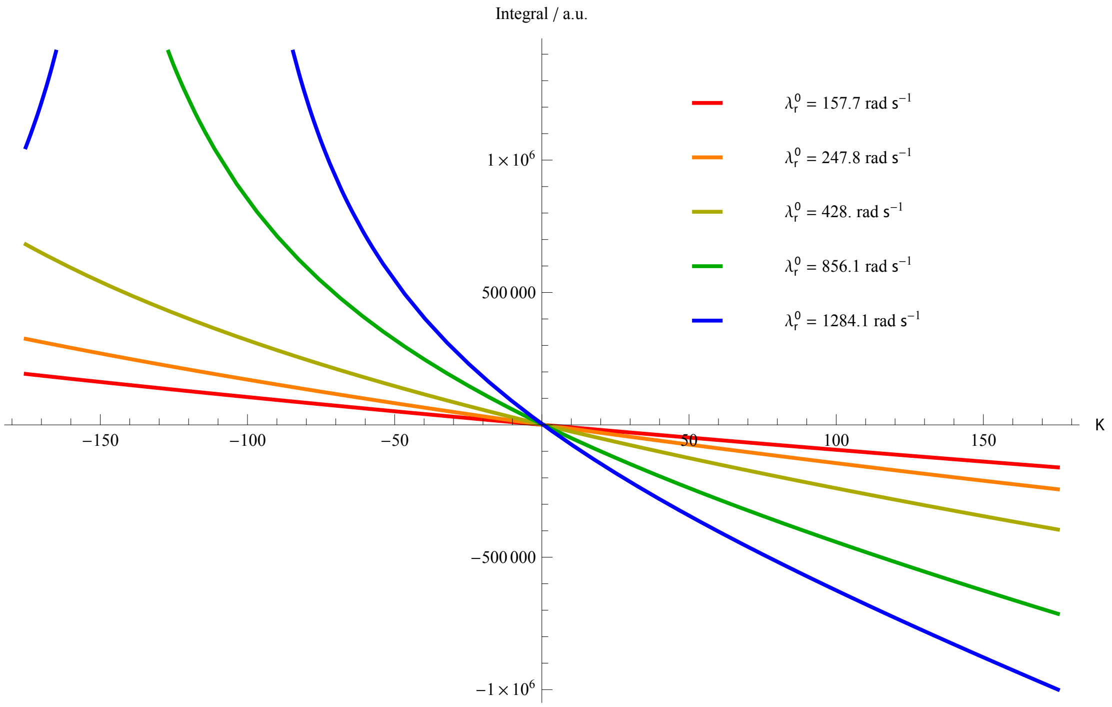

Figure S2: Spin noise integral calculated using Eq. (9) in the main text plotted as a function of K for a range of different thermal equilibrium radiation damping rates λ_r^0. The colors match with Fig. S1 and the corresponding entries in Tables S1 and S2.

The integral depends on K in a highly non-linear manner. Note that the cross-over points between positive and negative spin noise power integrals in Figure S2 do not occur at $K = 0$ but at an enhancement factor K_0 given by Eq. (S1).

$$K_0 = \frac{-\lambda_2 + \sqrt{(\lambda_2)^2 + \lambda_2 \lambda_r^0}}{\lambda_r^0} \qquad \text{(S1)}$$

The distances of these points from the origin are however not resolved at the scale used in Fig. S2. Thus, for larger values of λ_r^0, negative polarization levels result in a disproportionately large spin noise integral. As a consequence spin noise response for negative nuclear polarization exceeds the one for positive polarization as illustrated by the experimental spectra in Fig. 2 of the main text.
